# Contrast-noise-ratio (CNR) analysis and optimisation of breast-specific gamma imaging (BSGI) acquisition protocols

**DOI:** 10.1186/2191-219X-3-21

**Published:** 2013-03-25

**Authors:** Dennis Dieckens, Jules Lavalaye, Leo Romijn, Jan Habraken

**Affiliations:** 1 Department of Medical Physics, St. Antonius Hospital, , Nieuwegein, 3435, CM, The Netherlands; 2 Department of Nuclear Medicine, St. Antonius Hospital, , Nieuwegein, 3435, CM, The Netherlands

**Keywords:** Breast cancer, Scintimammography, ^99m^Tc-sestamibi, Breast-specific gamma imaging, Contrast-detail phantom, CNR analysis

## Abstract

**Background:**

Breast cancer is one of the most prevalent forms of cancer in women. Breast-specific gamma imaging (BSGI) is a diagnostic imaging method that uses sestamibi-labelled ^99^Tc and a dedicated gamma camera to localize malignant lesions in breast tissue. The aim of this study is to investigate if the current acquisition protocol for BSGI at our hospital is optimized for the detection of lesions in our patients.

**Methods:**

We analyzed patient data and performed a phantom study with a Dilon 6800 gamma camera. The patient data were collected from a group of 13 patients (740 MBq ^99m^Tc-sestamibi, four views per patient were dynamically acquired with a frame duration of 30 s per frame and a total acquisition time of 8 min per view). Reduced-time static images were created, and contrast-to-noise ratios of identified hotspots were determined for different acquisition times. For the phantom study, we used a contrast detail phantom to investigate the contrast and resolution properties, within the range of relevant clinical acquisition parameters. The phantom was filled with a concentration of 80 MBq in 500 ml of water, and we dynamically acquired frames for a total acquisition time of 60 min using a general purpose (GP) collimator. To compare the GP collimator with the high-resolution collimator, a second acquisition was made for both collimators with a total acquisition time of 16 min.

**Results:**

The initial analysis of BSGI scans of the 13 patients showed that a dose reduction by a factor of 3 would not have reduced the number of observable hotspots in each of the acquired views. However, a subsequent systematic analysis of our protocol with a contrast-detail phantom showed that dose reduction results in a lower observability of hotspots, whereas increased doses resulted in a higher observability.

**Conclusion:**

We believe that the results of our phantom study are relevant for clinical practice and that further dose reduction cannot be recommended for the BSGI exams at our hospital and that an increase of the administered activity should be considered.

## Background

Breast cancer is one of the most prevalent forms of cancer in women. In the Netherlands, every year 13,000 new cases are reported, and it is a condition that affects one in every nine women. Patients with breast cancer have a good prognosis if the disease is detected early. Mammography is the standard modality of choice for screening and diagnosing breast cancer. Unfortunately, the mammogram is not conclusive in all cases, especially in women with dense breast tissue; mammograms can be difficult to interpret and can lead to ambiguous findings. As a result, these patients are referred for a magnetic resonance imaging (MRI) scan. Breast MRI has a high sensitivity for detecting malignancy in the breast, but specificity is sub-optimal, potentially leading to unnecessary invasive procedures [1]. Breast-specific gamma imaging (BSGI) is an improved breast scintigraphy technique that uses a sensitive single-head small-field-of-view gamma camera [2-4]. Patient dose minimisation is an important aspect of the optimisation of any nuclear medicine protocol. The importance of dose optimisation depends, among others, on the patient population. For population screening, a much lower patient dose is acceptable than that for a patient group with a high risk of malignancy because in the screening setting, the number of subjects is higher and the chance of an individual positive finding is much lower. The average mean glandular radiation dose for a two-view digital mammography is 3.7 mGy [5], from which we estimate the patient dose (using ICRP 103 using a weighting factor of 0.12) for a four-view mamography exam to be 0.88 mSv. For a typical BSGI examination, the patient dose varies between 5.9 and 9.4 mSv for an administered activity of 740 to 1,184 MBq of ^99m^Tc-sestamibi. Recent work by Hruska et al. showed that significant dose reductions could be achieved when a dual-head breast-specific gamma camera is used [6,7]. At our hospital, we have a single-head breast-specific gamma camera (Dilon 6800, Dilon Technologies Inc., Newport News, VA, USA) for which we want to explore the possibilities of reducing acquisition times or, equivalently, further reducing patient dose. Specifically, this study concentrates on dose optimisation of the BSGI protocol at our hospital, and we introduce a contrast-to-noise ratio (CNR) analysis methodology and a phantom-based approach to determine if imaging longer (or equivalently giving a higher dose) could lead to the observation of more hotspots. We also applied the CNR analysis to data from a small group of patients.

## Methods

### Patient data acquisition

The acquisition protocol at our hospital consists of a single dose of 740 MBq of ^99m^Tc-sestamibi (5.9 mSv) that is administered intravenously to the patient. On average, the acquisition of images started 22 min p.i. Subsequently, craniocaudal (CC) and mediolateral oblique (MLO) views are acquired for each breast. The four views (RCC, RMLO, LCC, LMLO) are acquired with a general-purpose collimator for 8 min per view using a matrix size of 80×80 pixels and a pixel size of 3.2 mm × 3.2 mm. Our acquisition protocol has been adapted from the scintimammography protocol of the Dutch Society of Nuclear Medicine (NVNG) and is in line with the guidelines of the SNM [8] and the EANM [9]. For this study, we analyzed data from 13 patients (all patients were female, age range 43 to 66 years; mean 50.9 years) who were all referred to our department because of known or suspected malignant disease. The patient exams were performed using a dynamic acquisition mode, with a frame duration of 30 s per frame and a total acquisition time of 8 min. From the acquired data, we determined the background count rates, hotspot contrasts and hotspot sizes for different acquisition times. From the acquired views, a total of 49 views were included in the study (one patient had previously undergone a breast resection, and for another patient, the acquisition time from one of the views was reduced to 5 min due to patient discomfort). We derived time-reduced static images, from the dynamic data, for acquisition times of 2,2.67,4 and 8 min. The 8-min static views were used for routine diagnostic purposes. We used the reports from the nuclear medicine physicians as the reference from which we identified all the clinically relevant hotspots for which we determined the CNR.

Based on the retrospective nature of this study, IRB was waived by the Medical Ethics Review Committee of St. Antonius Hospital.

### Contrast-detail phantom and acquisition

**Figure 1:**
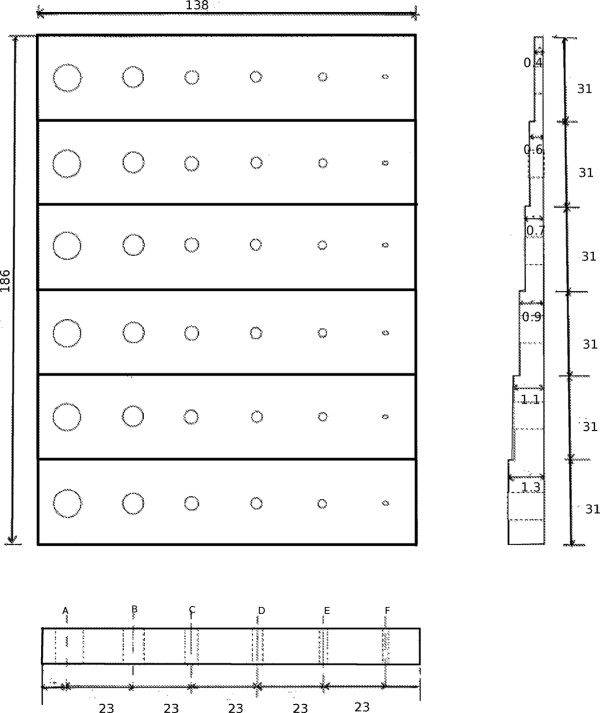
**Contrast-detail phantom.** Schematic of the contrast detail phantom design. The dimensions of the phantom are 186 mm × 138 mm. Within each row, the separation between the centres of the holes is 23 mm. The separation between the centres of the holes in adjacent rows is 31 mm. The depths of the holes in each row are 1.3, 1.1, 0.9, 0.7, 0.6 and 0.4 cm, respectively.

To simulate the hotspots that we see on the scintimammography images in clinical practice, we used a contrast-detail (CD) phantom which shows ‘ hot ’ areas or lesions of varying sizes and contrasts in known locations in a background that has no cold spots. The contrast-detail phantom that we used was built at the department of biomedical engineering at our hospital, based on the design previously published by Moré et al. [10]. In Figure 1, we show a transverse view of the design of the phantom.

**Figure 2:**
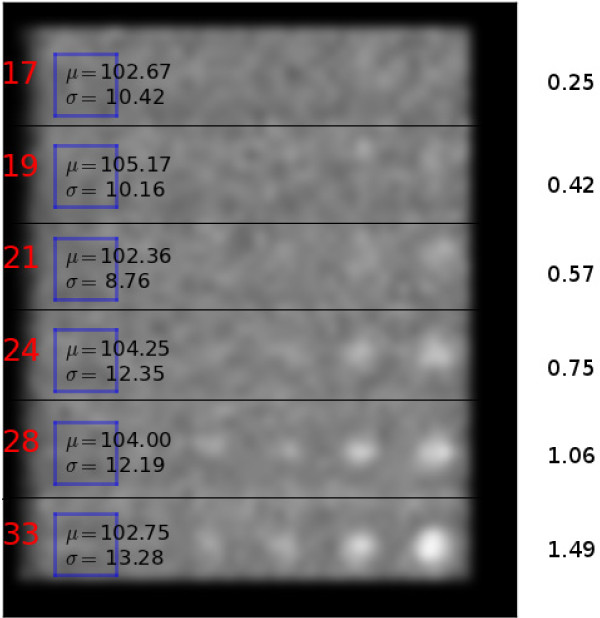
**Structure of composite normalized image. **Example of a composite normalized image. The numbers on the right show the expected contrast of the holes in each row based on Equation 1 and are also summarized in Table 1.

The phantom was constructed using polymethylmethacrylate (PMMA) and consists of six different rows with varying thicknesses of 1.3, 1.1, 0.9, 0.75, 0.6, 0.4 mm, respectively, and each row having six holes with 2, 3, 4, 5, 7.5 and 10 mm diameter, respectively. The CD phantom covers a range of contrasts, but the interpretation of the acquisition data of the phantom is complicated by the fact that for a fixed acquisition time, hotspots of the same size in different rows not only have different contrasts, but also have a different background per row. Clinical images only show hotspots of varying sizes and contrasts in a fixed background. We, therefore, processed the dynamic data obtained from the phantom to obtain a homogeneous background for all holes of the phantom in the following way: After the dynamic acquisition of the data, we identified the six rows of the phantom in the image. Next, we created a composite normalized image of the original data. This was done by selectively summing frames, in such a way that the resulting image has the same background everywhere. This is illustrated in Figure 2, where to produce a homogeneous background, the number of summed frames is different for each row: 17,19,21,24,28 and 33, respectively. The statistics of the background regions of interest (ROIs) are shown in Figure 2, and we see that the image has a homogeneneous background (on average 103.5 counts/pixel with a standard deviation of 1.1 between the rows).

The contrast of the holes in each row of the phantom depends, in first approximation, on the thickness of the PPMA and is given by the following equation [10]:

(1) $$C=e^{\mu_{a}(h-l)}\left[\frac{1-e^{\mu_{W}h}}{1-e^{\mu_{W}l}}\right]-1,$$

where *μ*_*a *_(0.17 cm ^-1 ^for 140 keV) is the linear attenuation coefficient of accrylic, *μ*_*w *_(0.15 cm ^-1 ^for 140 keV) is the linear attenuation coefficient of water, *h *is the height of the column for each hole, and *l *is the height of the background columns. The contrast, as described by Equation 1, is independent of the diameter of the holes and of the acquisition time. In other words, the background counts are controlled by the acquisition time, but the contrast will remain the same in principle. In Table 1, the theoretical contrasts of the phantom are given including the attenuation of the PMMA and excluding the partial volume effect.

**Table 1:** Theoretical contrasts of the phantom

**Row**	***l***	**Contrast**
1	1.1	1.49
2	1.3	1.06
3	1.5	0.75
4	1.65	0.57
5	1.8	0.42
6	2.0	0.25

Overview of contrasts based on Equation 1 for each row.

For the measurements, we filled the phantom with a concentration of 80 MBq of ^99m^Tc in 500 ml of water. The phantom was subsequently imaged with a general-purpose (GP) collimator and a high-resolution (HR) collimator. These collimators have different resolution and sensitivity properties, with a sensitivity of 180 and 90 counts/MBq for the GP and the HR collimator, respectively. The ratio of these sensitivities numerically equals 2.0, which means that to receive the same number of counts, one needs to image twice as long with the HR collimator compared to the GP collimator.

**Figure 3:**
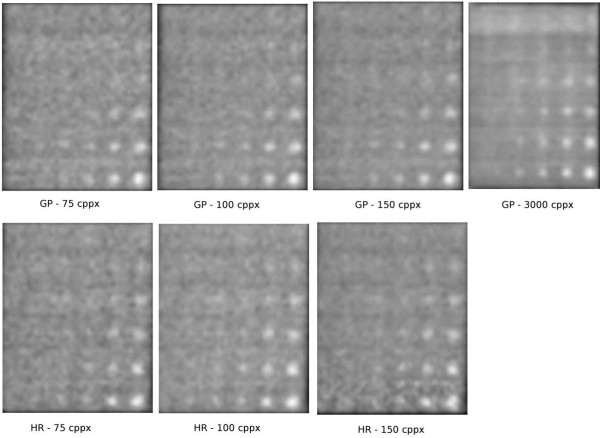
**Examples of acquired GP and HR images. **Example of normalized phantom images for data acquired with both GP and HR collimators.

The acquisition of the filled phantom was performed using a dynamic acquisition mode with a frame duration of 30 s for a total acquisition time of 60 min, using a GP collimator. To compare the GP collimator and the HR collimator, a second acquisition was made for both collimators with a total acquisition time of 16 min and a frame duration of 10 s per frame. In Figure 3, we show examples of the normalized phantom image for four different backgrounds for the GP and the HR collimator, respectively.

The orientation of the images is such that in the vertical direction, the contrast increases as we move down the rows. In the horizontal direction, the holes of the phantom become smaller from right to left, and due to the partial volume effect, the observed size of the holes levels off and, instead, the contrast becomes smaller.

### Analysis

The observability of a hotspot in a noisy background depends not only on the contrast of the hotspot, but also on the observed size of the hotspot and on its background. We therefore have performed a CNR analysis of both the patient data and the phantom data. The CNR is a dimensionless parameter and defined here as [11] follows:

$$\text{CNR}=\frac{C}{\text{COV}},$$

where the contrast C is defined with respect to a background region and given by *C *= (*N*_*l*_-*N*_bg_)/*N*_bg_, where *N*_*l *_can be either the maximum or average number of counts per pixel measured in the ROI that was drawn around the hotspot. Because most of the hole diameters are comparable to or smaller than the pixel size of the camera, we have used the maximum ROI value for our analysis. *N*_bg _is the average number of counts of the background. The background ROIs for each row were chosen to be as large as possible without overlapping with the holes in each row. The coefficient of variation (COV) is given by COV = *σ*_bg_/*N*_bg_, where *σ *_bg_is the standard deviation of the background ROI.

The detectability of a hotspot by an observer is correlated with the CNR value of the hotspot. The Rose criterion states that if the CNR of a hotspot becomes smaller than 3 to 5, it becomes very difficult to observe the hotspot[11]. By reducing the acquisition time, we determined the number of hotspots that have a CNR smaller than the Rose criterion. It is important to note that the CNR analysis of the patient data was performed on the CC and MLO views separately, which means that only conclusions about the detectability of a lesion on a separate view can be made. For the phantom, we have defined the observability as the percentage of the holes in the phantom that have a CNR greater than a given threshold value (based on the Rose criterion). For example, if the observability of the phantom is 50% with a threshold of 3, this means that 50% of the 36 holes of the phantom have a CNR_max _that is greater than 3. By determining the observability as a function of acquisition time, we can characterize the way in which the detectability of lesions increases with increasing acquisition time for a given camera. We repeated this procedure for a range of threshold values to determine the dependency on the threshold value. As an additional practical application of our method, we compared the observability of the GP and the HR collimator, taking into account the difference in sensitivity of the two collimators.

## Results

### Patient data

**Figure 4:**
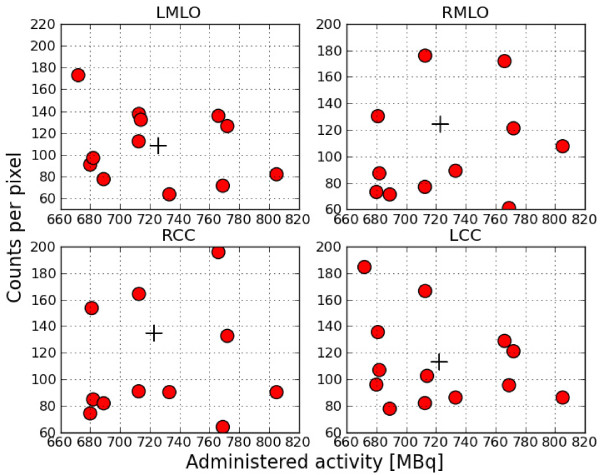
**Measured background counts in patients. **Overview of measured background counts versus administered activity after 8 min. The plus sign indicates the average activity and background counts.

To determine the average background counts, we manually drew ROIs on all views. This includes views with hotspots and views without hotspots. In Figure 4, we give an overview of the measured background counts versus administered activity after an acquisition time of 8 min for each of the views. The average background of the patients included in this study was 120 counts per pixel (range 64 to 195) after an acquisition time of 8 min with an average administered activity of 723 MBq.

**Figure 5:**
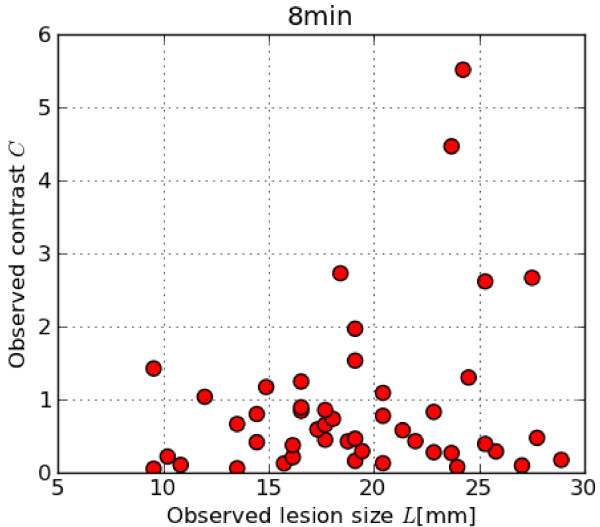
**Observed lesion sizes and contrasts. **Overview of observed lesion sizes versus observed contrast.

From the set of 49 views, the nuclear physician identified 52 hotspots to be possibly clinically relevant. Next, we determined the observed size and contrast of the hotspots with respect to the background of the image. The results are shown in Figure 5.

**Figure 6:**
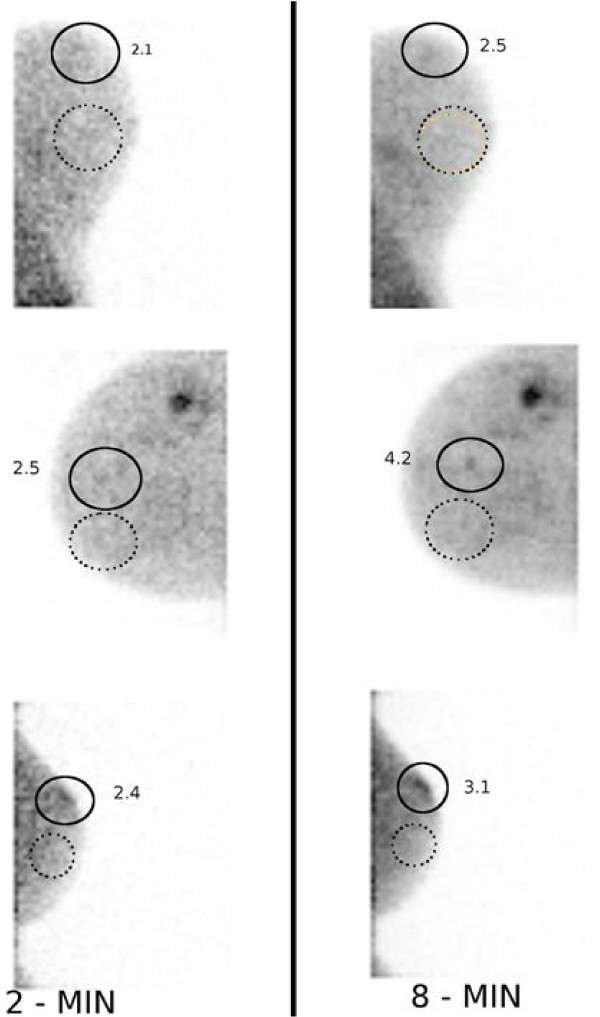
**Example of CNR measurement in patient data. **Three patients with hotspots for which CNR_max _≤ 2.5 when the acquisition time is reduced to 2 min. The dotted line corresponds to the background ROI used, and the solid line marks the hotspot of interest. The numbers shown correspond to the CNR_max _within the ROI.

From Figure 5, we notice that the smallest observed hotspots sizes are around 9 mm, and the lowest observed contrasts are around 0.1. The CNR values of the identified hotspots are summarized in Table 2 for the various acquisition times. We see that all indentified hotspots have a CNR_max _> 2.5 after the acquisition time of 8 min. For this reason, we use CNR_max _= 2.5 as our clinical reference value throughout this study. From Table 2, we see that when we reduce the acquisition time by a factor of 4, three of the identified hotspots have a CNR_max _< 2.5, and visual inspection confirmed that these hotspots were no longer observed. In Figure 6, we show the corresponding patient data for these three hotspots. We have marked the background ROIs (dotted lines) and the three hotspots (solid lines).

**Table 2:** Analysis of CNR data of patient data

**Scantime (min)**	**CNR**_**max **_ ***≤ 2.5***	**CNR**_**max **_ ***> 2.5***	**CNR**_**max **_ ***> 3***	**CNR**_**max **_ ***> 4***	**CNR**_**max **_ ***> 5***
2	3	49	46	38	33
2.66	0	52	46	38	33
4	0	52	44	38	34
8	0	52	49	39	35

Overview of CNR_max _for 52 hotspots at 2, 2.67, 4 and 8 min, respectively.

The hotspots which had both maximum and mean CNR<2.5 were not detectable when reviewed.

In summary, we see that for our patient group, the identified hotspots all have a CNR_max _> 2.5. Reducing the scanning time or, equivalently, patient dose by a factor of 3 does not lead to hotspots with CNR_max _< 2.5.

### Phantom data

**Figure 7:**
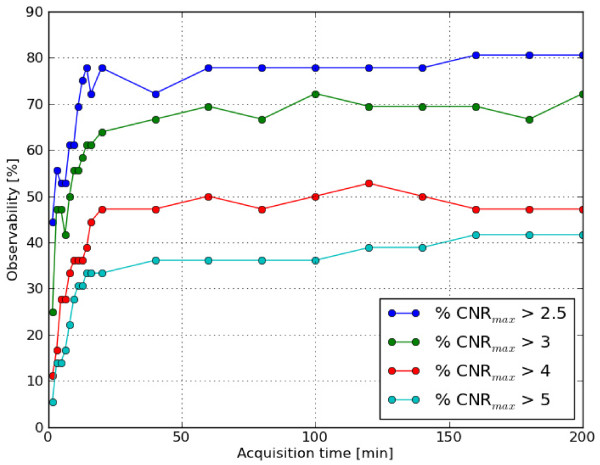
**Observability of the phantom. **The observability (see text for definition) of the phantom for a GP collimator versus acquisition time in minutes. The different lines correspond to different threshold values.

To compare the phantom data acquired with the GP collimator and patient datasets, we determined for the phantom data for each acquisition time the average number of background counts. The average background counts in the phantom were then converted to an equivalent patient acquisition time by using the fact that after an 8-min acquisition, the average patient has 120 counts/pixel in the background. To compare the HR and GP collimator, we additionally took into account the difference in sensitivity between the two collimators. In Figure 7, we show the observability of the GP collimator.

It can be seen from the figure that, independent of the threshold value, the observability increases until the acquisition time reaches 16 min and then levels off. The current protocol uses an acquisition time of 8 min, which lies right in the middle of the range where the observability increases in the phantom. This suggests that increasing the acquisition time of the protocol could result in a higher observability for patient acquisitions as well. As an additional practical application, we compared the observability of the GP and HR collimators.

**Figure 8:**
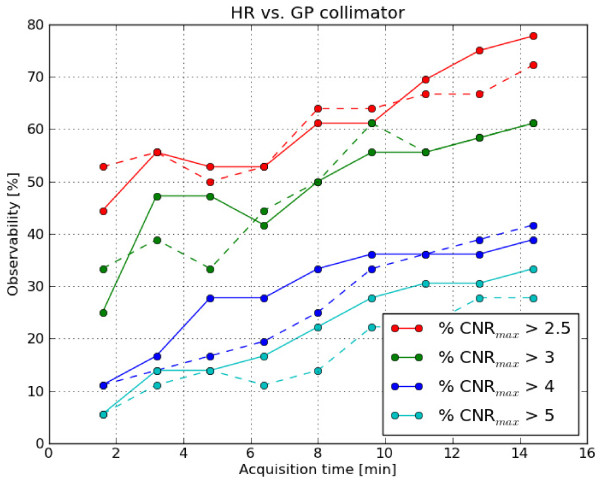
**Comparision of the GP and HR collimator. **Comparison of the observability of the phantom for the GP collimator (solid line) and the HR collimator (dashed line). The different colors correspond to different threshold values.

As shown in Figure 8, the observability of the HR and GP collimators is similar for each threshold value. To the extent that CNR can be used as a measure of image quality, this means that images acquired with the HR collimator have the same image quality as those acquired with the GP collimator at the same acquisition time.

## Discussion

In this study, we presented a CNR analysis of the BSGI acquisition protocol at our hospital. We performed the CNR analysis both on patient data and on data acquired with a contrast-detail phantom. Using a dynamic acquisition protocol, it was possible to create time-reduced images for any acquisition time shorter than the acquisition time of our current protocol. The results from the patient data analysis showed that the acquisition time could have been reduced by a factor 3 without reducing the number of observable hotspots on the separate views. Additional visual observation of the identified hotspots at the reduced acquisition times confirmed our finding. In contrast, the phantom study showed that increasing the dose or acquisition time resulted in an increased detectability of small lesions with low contrast. We believe that the explanation for the discrepancy between our findings in the patient and phantom data is the small number of patients included. If we include more patients we will eventually find tumours that can only be detected with confidence by an observer after the full acquisition time. The fact that we see many patients with relatively large tumours with a high CNR is related to the referral procedure for BSGI exams at our hospital. In most cases, patients who are referred for a BSGI examination at our hospital already underwent mammography, ultrasound and MRI exams, and there is often a strong suspicion for malignant disease; however, additional information about the size or focality of the disease are required. Furthermore, based on our phantom data, we cannot exclude the possibility that smaller lesions with low contrast were in fact present in the patients that we used for our data analysis and would have been detected using a longer acquisition time or higher dose but remained undetected with our current protocol.

To systematically investigate our findings of the patient data analysis, we used a contrast-detail phantom. The reason a contrast-detail phantom can be used to simulate clinical practice is because the detection of breast cancer always involves the recognition of hot areas or lesions in a background that have no cold spots. The contrast-detail phantom simulates hotspots with contrasts ranging from clearly visible to definitely invisble. From the results of the patient data analysis (see Figure 5), we also see that there is an overlap between the lowest contrasts seen in the patient data and the higher contrasts of the hotspots in the phantom. The way an observed contrast is realised depends on many factors such as the depth, size and uptake of the physical lesion. A physically small lesion deep inside the breast can result in the same observed hotspot as a more superficial and larger lesion with a lower uptake of radioactive tracer.

Due to the partial volume effect, the observed sizes of the smallest lesions are significantly larger than the true sizes of the lesions. The smallest observed size of hotspots from both the analysis of our patient data and from the phantom study was around 10 mm. For the phantom, we know that the true diameter sizes of the hotspots varied from 1 to 10 mm. For the patient data, we could not correlate the physical size of the lesions with the observed hotspot sizes because we did not have the final pathology reports available. It should be noted that in the literature, tumours as small as 1 mm have been reported to be detected with BSGI [3]. The smallest hole that we were able to observe in this phantom study was 2 mm.

**Figure 9:**
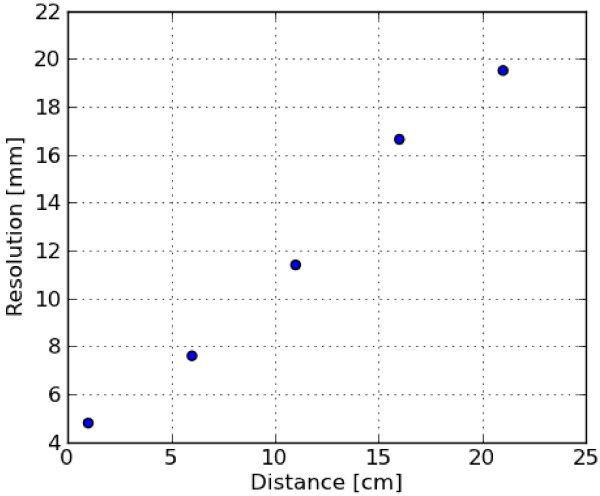
**Resolution versus distance. **The FWHM resolution as a function of the distance to the detector.

As mentioned, the contrast-detail phantom we used does not simulate lesions at different depths in the breast, and although this would give additional and possibly valuable information, we believe this is less important for this study, where we were able to show that hotspots with lower contrasts become visible if we image longer. We have also demonstrated that some holes will remain undetectable by an observer, even after extremely long acquisition times. It would therefore be interesting to repeat the phantom measurements and analysis on different BSGI systems. To get an idea of the depth dependence of the system resolution, we measured for our camera (see Figure 9) how the system resolution changes as the distance to the detector increases. For the clinically relevant range of compressed breast sizes, the resolution decreases from 4.8 mm full width at half maximum (FWHM) at the detector to 7.6 mm at a distance of 6.5 cm. When we compared the resolution of our BSGI system with the standard gamma cameras at our institute, we found that the resolution of the BSGI system deteriorates faster with increasing distance, but for clinically relevant distances of up to 8 cm, these differences remained relatively small. Besides the loss in resolution, deeper lesions will have a decreased CNR due to scatter and attenuation.

Although the phantom is not an anatomical (3D) model of a breast, it is a good simulator for (planar) scintimammography images. The phantom we used is easy to manufacture and easy to operate. Analysing the phantom data required some reformatting of the data. From the phantom study, we see that the optimal acquisition time is around 16 min. In practice, long acquisition times are undesirable. Arguably, acquisitions lasting 8 to 10 min per view are reasonable. Longer acquisition times will also likely introduce additional artefacts due to patient movement. As a result, too long acquisition times are clearly impractical, and somehow an upper limit to the acquisition time per view will have to be introduced. This means that a higher sensitivity cannot be achieved by longer acquisition times, and the only practical solution to increase the chances of detecting more lesions for our system is to increase the patient dose. Alternatively, a dual-headed system has a greater sensitivity and should lead to improved detectabillity as well. The CNR analysis can be used on the patient data as well as on the phantom data with the benefits of reproducibility, consistency and objectivity. As mentioned above, the Rose criterion makes a connection between the CNR value of a lesion and the probability of observing the lesion. The Rose criterion, however, does not refer to a single threshold value but to a range of values that separates lesions that can be observed with near certainty from lesions that are almost certainly unobservable. In this study, we showed that for each threshold value in the range of 2.5 to 5, the observability of the phantom levels off at the same acquisition time.

As an additional practical application, we compared the observability of the GP and HR collimators and found that the observability of the HR and GP collimators is similar for each threshold value and acquisition time. It should be mentioned that there are also dual-headed breast-specific gamma cameras based on CZT technology, whose scanning technique is equivalent to the single-head configuration. The dual-headed cameras have an even greater sensitivity than the single-headed configurations, and a proof of concept evaluation in patients using 296 MBq has indicated that further dose reduction seems feasible [6,7]. The downside of the dual-headed cameras is the significantly higher cost, and the modality cannot be moved around as easily. To quantify the differences between the single- and dual-headed cameras, it would be interesting to compare the two modalities directly using the CD phantom and the CNR analysis presented here.

## Conclusion

In summary, we found that although a dose reduction by a factor 3 in an initial study of 13 patients would not have lead to a reduced number of observable hotspots in each of the separate views acquired, our phantom study showed that a further dose reduction of BSGI scans of 8 min/view with 740 MBq might result in a reduced sensitivity for lesion detection, whereas increasing the acquisition time and/or activity might result in a higher sensitivity for lesion detection. At our hospital, BSGI is used for a patient group that has a high incidence of malignant disease, and the main clinical question is to characterize the extent and focality of the disease because this has important consequences for the course of treatment. An increase of the administered activity might therefore be justified, although a detailed radiation risk analysis should be performed to determine how much the activity might be increased to achieve an optimal detection possibility of small lesions in our patient group.

## Competing interests

The authors declare that they have no competing interests.

## Authors’ contributions

DD, JH and LR designed the study. JL supervised the clinical part of the paper. All authors edited the manuscript. All authors read and approved the final manuscript.
